# Ophthalmological aspects of mustard gas poisoning (focus on management)

**DOI:** 10.22088/cjim.13.3.458

**Published:** 2022

**Authors:** Mehrdad Rafati-Rahimzadeh, Mehravar Rafati-Rahimzadeh, Sohrab Kazemi, Seyedeh Roghieh Jafarian Amiri, Abbas Soleymani, Ali Akbar Moghadamnia

**Affiliations:** 1 Department of Nursing, Babol University of Medical Sciences, Babol, Iran; 2 Neuroscience Research Center, Health Research Institute, Babol University of Medical Sciences, Babol, Iran; 3 Department of Medical Physics, Kashan University of Medical Sciences, Kashan, Iran; 4 Cancer Research Center, Health Research Institute, Babol University of Medical Sciences, Babol, Iran; 5 Department of Ophthalmology, Babol University of Medical Sciences, Babol, Iran; 6 Cellular and Molecular Biology Research Center, Health Research Institute, Babol University of Medical Sciences, Babol, Iran

**Keywords:** Sulfur mustard, Mustard gas, Blistering agents, Alkylating, keratoplasty, Tarsorrhaphy, Corneal transplantation

## Abstract

**Background:**

Amongst the chemical warfare agents, blistering (vesicant) agents can be significant materials. The most important agent in this group is sulfur mustard (mustard gas) which is known as “King of chemical warfare (CW) agents “. Exposure to this agent, seriously causes damages in several organs, such as the eyes. This article reviews the ophthalmological aspects of sulfur mustard with reference of its management.

**Methods:**

A wide-ranging search in PubMed databases, Thomson Reuters and Scopus was done and different aspects of chemical properties of sulfur mustard, its mechanism of action and effects on eyes, clinical finding, diagnostic evaluation, initiate actions, pharmaceutical and surgical interventions was reported.

**Results:**

Sulfur mustard can alkylate DNA and RNA strands and break down structures of protein and lipid of cell membrane. This may impair cell energy production, and leads to cell death. Exposure to sulfur mustard, therefore, causes such problems for organs, including irreversible damage to the eyes.

**Conclusion:**

Understanding the mechanism of the sulfur mustard effect and the early training in prevention injuries will cause fewer complications and damage to organs, including the eyes. Washing the eyes with tap water or eyewash solutions, using mydriatic drops, anti- inflammatory drugs, matrix metalloproteinase inhibitors and antibiotics may help to the management of poisoning. Surgical interventions including tarsorrhaphy, amniotic membrane transplantation, stem cell transplantation and corneal transplantation could reduce the harm to the victims.

From decades ago till now, because of the copious access to chemical compounds, the poisoning has spread amazingly in the world (1, 2). People are usually poisoned accidentally or intentionally because of the misuse or excessive use of these chemicals (3, 4). The importance of the matter is the use of these chemical agents by terrorists or in local-regional wars. This causes short and long –term injuries, incapacitates, kill ordinary people in war affected areas and troops in war zones (5). The first reports of using chemical warfare agents started in ancient Greece and Rome. The warfare had been used in World War I and repeatedly used up to now (5, 6). One of the most well-known destructive substances is chemical warfare agents. Chemical warfare agents are one of the most known mass destructive materials. In the past decades, these agents have been used many times with different classes and chemical properties. They cause inability, death, or long term suffering of contaminated people (7). 

Sulfur mustard is a vesicant (blistering) chemical warfare agent which is known as “King of Chemical Warfare (CW) agents”. Other vesicant (blistering) agents with different mechanisms include nitrogen mustards(HN1, HN2, NH3) and lewisite (L1,L2,L3) (8). The most important cause of blisters is sulfur mustard or mustard gas, which is also called Y-perite (a place in Belgium), the yellow cross ( assign that the Germans put on people's clothing) ,and even the word LOST (the first family name of two German chemists) (9). First, German military forces used sulfur mustard in the battlefield near Y- perite (Belgium) in July 1917. Afterwards, in December 1943, an allied forces ship containing sulfur mustard exploded at the port of Bari, Italy. All of the chemicals were spread around and caused 600 deaths (10). They had been used sporadically since World War I (11), but the latest military use of this material was in Iraq war against Iran by Iraqi forces (1980-1988).The peak use of this warfare was in Halbja in 1988 by Iraqi fighters bombing , which over 100000 victims were damaged in this chemical attack. and one-third of the people suffer from their late effects, so far (9, 10, 12).

■ **Physicochemical properties: **Impure sulfur mustard smells similar to mustard or garlic, but its pure sulfur mustard (C_2_H_4_Cl_2_S) is colorless and odorless. It is quickly soluble in organic solvents but very little soluble in water (7). Its chemical characteristics include: molecular weight; 159.08, specific gravidity; 1.27, freezing point; 14.45 °C and boiling point; 215-217 °C (9). It is a strong alkylating, nucleophilic, strong lipophilic (very hydrophobic), cytotoxic agent with mutagenic and carcinogenic properties (5, 13).

■ **Mechanism of action: **There are several theories about the mechanism of action of sulfur mustard. The first one is acid producing action which is based on hydrolysis sulfur mustard to hydrochloric acid within cells. But vesicant action cannot do to the content acid release, this theory was rejected (9, 14, 15). The second is the changes in sulfur with proteins and several inhibited enzymes, particularly hexokinase. Importantly, the level of alkylation for inhibition enzymes in vitro did not watch the vesicant dose in vivo, in fact it is not enough. In addition, as a result of alkylation, hexokinase is inhibited after a few minutes. But tissue damage is shown by sulfur mustard in vitro and in vivo. Therefore, this theory was also rejected (9, 14, 15). The third theory states that sulfur mustard can reduce the level of cellular glutathione and induces lipid peroxidation. Based on this mechanism, sulfhydryl containing compounds (e.g. proteins and peptides) are quickly deactivated. Sulfhydryl groups protect compounds from oxidation in cells. In fact, when sulfur mustard contacts with cells, lipid peroxidation and glutathione depletion may occur. These events are mediated by the reaction of oxygen species. It is important to note that not all of the above theories are in consistent with the delay damage by sulfur mustard. However, some theories confirm the toxicity of sulfur mustard (9, 10).

The most important mechanism proposed is alkylation of cell components by sulfur mustard. This means that alkylation reactions occurred with cell components essentially protein, lipid membranes, as well as DNA and RNA. It automatically assumes intramolecular cyclization. The elimination of chloride ion and formation of ethylene sulfonium ring are caused by this cyclization. Sulfonium ion can cause alkylation of sulfhydryl (-SH) and amino (-NH2) groups. For example, such a mechanism makes indirectly inhibition of glycolysis (16). In the middle process, ethylene sulfonium is converted to carbonium ions and reacts immediately with DNA, RNA, protein and other available molecules(9). Finally, sulfur mustard reacts with the DNA, and alkylation occurs at the N7 position of guanine (7- (2-hydroxyethyl ethyl ethyl)) guanine (7-HETE-G), the N1 and N3 positions of adenine, and the O6 position of guanine) (17, 18).

Single and double strand DNA breaks can be induced by DNA alkylation. Following this process, the cells attempt to correct the loss to the DNA. This process activates poly(ADP-ribose) polymerase(PARP), whose two classes,PARP-1 and PARP-2 are activated by breaking the DNA strand(19). As a first- line protein (PARP-1), this will counteract DNA strand breakage. This plays a role in continuing survival and integrity cell against genotoxic stress(20). It should be noted that the extreme activity of PARP leads to deplete of nicotinamide adenine dinucleotide (NAD+), in addition to decrease in glycolysis at the same time(15). The results of depleting NAD+ and preventing glycolysis conduct to a defect in energy production in the cell and loss of ATP and eventually cell death (17).

■ **Clinical Manifestation**

● **General: **Often in the first stage of exposure to sulfur mustard, we will not confront significant complications. Naturally, the symptoms appear after several hours, before that only the smell of garlic or sulfur, and the maximum symptoms reveal after a few days. The organs that will be most affected in contact with sulfur mustard are eyes, respiratory system and skin, in addition signs and symptoms are acute or chronic (9, 16, 21). If sulfur mustard is spread in the air in a battlefield, it imposes damage on the organs mentioned above. Also, various factors will play in the severity of the injury, such as dosage, exposure way, individual characteristics (age, sex, height), protection methods, environmental factors (heat humidity), and other factors (15). The dosage amount and its effects on the body are as follows; in humans, a lethal dose of 200 mg orally, in bare skin that has been exposed to sulfur mustard for a long time, 4-5 grams, and in the respiratory system 1500 mg per minutes per cubic meter, and the exposure time(minutes, hours, days, or weeks) (22).

● **Clinical Manifestation of Eyes: **The eyes are the most important organ that can be damaged by contact with sulfur mustard. Degree of eye damage is varying, and it is estimated that the damage is seen about 50 to 90 percent of the victims. Even small amounts can cause visual impairment and disability. The sulfur mustard concentration threshold for eye damage is 12 mg/min/cubic meter and for skin is 200 mg/min/cubic meter (23, 24). In the first hour after edema and conjunctivitis, symptoms such as grittiness (feeling sand in the eyes), bloodshot eyes (redness of the eyes), and the development of edema occur. Inflammation of the cornea and simple conjunctivitis occur in amount of less than 50 mg/min/cubic meter and edema occur in more than 200 mg/min/cubic meter (24). Feeling severe pain in the eyes, laceration, photophobia, blepharospasm and reduced visual acuity after 2 to 6 hours, also after 12 hours the patient poses with temporary and transient blindness, and spontaneous recovery will occur after 48 hours, but complete corneal regeneration of the corneal epithelium is seen after 4 to 5 days, although complete recovery requires 6 weeks and even more. It is important to note this point, that damage to the cornea can lead to scar, cellular necrosis and long term complications(24, 25).

Irreversible visual impairment and even blindness are the delayed and severe findings, although this is controversial. Corneal epithelial failure and the formation of neovascularization in the cornea, thinning and blurriness in laboratory animals and humans are seen for a few weeks and several years , respectively (23, 26). A transient increase in intraocular pressure with low grade iridocyclitis without synechia or cataract formation may be seen after a few days (22, 27, 28). 

Long- term complications of sulfur mustard exposure are chronic or delayed- onset keratitis. It may be penetrating keratoplasty (29). Victims of keratitis problems suffer from impaired corneal sensation, recent and continuous corneal erosion, fatty and amyloid deposition, irregularity, thinning and corneal ulcers, limbal stem cell deficiency (mild to moderate), and neovascularization in the cornea (15, 22). Limbal epithelial stem cell deficiency and dysfunction of limbic stroma are due to destruction. Limbal stem cell deficiency affects all quadrants, especially the nasal and temporal regions (30). In table 1, two factors, the dose in milligrams per minute per cubic meter and the time in hours, days, and weeks in eye injury, are indicated for exposure to sulfur mustard (31, 32).

**Table 1:** The patterns of ocular damage as a result of sulfur mustard exposure

**Phase**	**Severity**	**Ocular disorders**		**Dose in mg/min/m** ^3^ ** (environment air)**	**Duration **
		**Symptoms**	**Signs**		
Acute	Mild	Foreign body sensation, tearing, photophobia, blepharospasm,	Eyelids hyperemia, vascular dilation and hyperemia of the conjunctive	12-70 (In some cases, more than 100 to 200)	Up to 2 weeks
	Moderate	Same as mild damage, dry eye sensation, eye pain	Same as mild damage, conjunctival edema, corneal epithelial edema, corneal epithelial erosion, superficial punctuate keratopathy (more in the lid fissure area)		
	Severe	Same as mild and moderate, severe ocular pain, swelling, redness, sores and spasms of the eyelids, reduced vision	Same as mild and moderate, inflammation, edema and in some cases, secondary infection of the conjunctive, Ischemia and necrosis of the conjunctive, limbal ischemia and necrosis, corneal epithelial irregularity and defect, corneal stromal edema, possible corneal infection, inflammation of the anterior chamber (uveitis), perforation of the cornea		
Chronic and Delayed	Mild	Photophobia, burning, foreign body sensation in eyes, dry eye, tearing, slight redness of the eye	Meibomian gland dysfunction, chronic blepharitis, reduced thickness of the tear meniscus layer, telangiectasia of the conjunctival blood vessels, comma shape vascular tortuosity in the palpebral fissure area (nasal and temporal), subjunctival fibrosis, subconjunctival hemorrhage,conjunctival scarring, punctuate epithelia erosions	100-200 (In some cases, more than 200)	3-6weeks
	
	Moderate	Same as mild damage, reduced vision, marked red eye, itchy eyes, ocular pain	Same as mild damage, corneal irregular astigmatism, periods of relapse and remission, mild to moderate limbal ischemia, irregular cornea, thinning of corneal periphery, corneal opacity as well as lipid and amyloid material and deposition in the corneal periphery, peripheral corneal vascularization, peripheral stromal scars of the cornea, peripheral intra-corneal hemorrhage, transparency of the corneal center, decreased corneal sensation		
	Severe	Same as mild and moderate, severe photophobia, severe vision loss, severe pain	Same as mild and moderate, severe limbal ischemia, limbal cell deficiency, thinning and opacity of the central and peripheral parts of the cornea, corneal opacity as well as lipid and amyloid deposition in the cornea, central and peripheral corneal vascularization, band keratopathy and scars in the central and peripheral corneal stroma,central and peripheral intra-corneal hemorrhage, corneal conjunctivalization, corneal descemetocele, corneal ulcer, corneal melting and perforation, history of limbal and corneal surgeries		
		Permanent blindness		> 200	Very rare

■ **Laboratory Diagnostic Tests: **Urinary metabolites of sulfur mustard are appropriate for determining its contamination, but the problem is their short half-life. Proteins, as macromolecules, play an important role as a long-term biological marker, because they are detectable for a few months (33, 34). Sulfur mustard contamination can be detected through urine, blood, and blister exudates (34, 35). Diagnostic methods are as follows:

● **Diagnosis of urinary metabolites of Sulfur Mustard: **In this method with collection of thiodiglycol (TDG) (free and conjugated form) and its oxide TDG (TDGO) (free and conjugated form) is evaluated in urine. In fact, free TDG, free + conjugated TDG, free TDG+TDGO, and free plus conjugated TDG +TDGO should be collected for diagnosis of metabolites of sulfur mustard in urine. These samples are analyzed by liquid chromatography-mass spectrometry (LC-MS) and gas chromatography-mass spectrometry (GC-MS) methods (34, 36). Today, LC-MS-MS with electrospray ionization (ESI) detector has been introduced for the rapid analysis of beta-lyase metabolites. This method has replaced GC-MS-MS method, because it avoids change in metabolites to more volatile analyses and less polar (37).

● **Diagnosis of Sulfur Mustard adducts with DNA: **If the analysis of sulfur mustard poisoning is done in the long time, it is necessary to check the DNA and protein adducts, which is some good biomarkers. Due to the high affinity of DNAs with alkylating agents, the cytotoxicity of sulfur mustard is evaluated with good clear and accuracy (35). To reveal DNA- sulfur mustard adducts, the enzyme-linked immunosorbent assay (ELISA) is used (38).

● **Diagnosis of Sulfur Mustard adducts with proteins: ** As mentioned, for a long time, the study of metabolites in the urine is not appropriated, as DNA and proteins adduct will be used (sulfur mustard- proteins adduct in the paragraph above). The following is a discussion of the sulfur mustard and proteins adduct. Meanwhile, hemoglobin and albumin are easily separated to assign sulfur mustard adducts (10). Prominent adducts include six various histidine residues, glutamic acid residues, and both of the N-terminal values. The highest numerous is related to N1 and N3 histidine adducts and alkylated N-terminal valine adducts are the most beneficial for subsequent measurement (39). LC-MS-MS method is used to evaluate the formation of adducts (40).

■ **Treatment**

● **First aid measures, triage, general and antidotal treatment: **Victims should be carried as soon as possible from the contaminated area (hot zone) to decontaminated area (warm zone). All clothing of victims must be removed and people must be keep away from contaminated zones. Then, washing the skin with water and neutral soap (pH near 7) is necessary. It is important to note that the skin should not be rubbed and cleaned with a cloth, because it increases the absorption of sulfur mustard. When residence and settlement's area for victims is contaminated with liquid sulfur mustard, it should be noted that the vulnerability of eyes are high, it must be washed with a large volume of water, normal saline or ringer solution. The victims are then transferred to medical or safe places (41, 42).

After releasing of chemical battle agents in environment, the treatment team implement the plan for triage, they try to prioritize resuscitation, decontamination, drug treatment and transfer to hospital or safe area. Triage is a dynamic and fast operation that is always performed between the contaminated areas (hot zone) to decontaminated area (warm zone). Triage consists of four stages; T1(immediate or urgent stage): the stages of medical care and advanced life support for the victims in short time at the scene the accident to the hospital.T2 (delay stage):the victims have severe injuries that require extensive care and will require hospitalization, but delay in this care has no effect on the diagnosis of the accident.T3 (minimum stage): the victims have suffered minor injuries that do not require leaving the place of residence and will return to their duties in short time.T4 (expectant stage): the victims have severe and fatal injuries and with the existing care, the probability of living them is low, they die before reaching the terminal care (42). To facilitate the classification of triage and activity of the treatment team and rescuers, and the important in the combat zone, four colors have been adopted; red for the immediate or urgent stage, yellow for the delayed stage, green for the minimal stage and black the expectant stage (43).

In clinical setting, a sedative is used to control pain and relaxation (33). No antidote for poisoning with sulfur mustard has been confirmed. Although it is recommended as an antidote to prevent side effects of alkylating agents, no drug has been approved in humans for antidote administration (5).

**◘ Eye lesions management:**

**1) General measurement and medical treatment**

Eyes exposure to sulfur mustard is a significant concern for the treatment team. It causes damage to the eyes and causes incapacitating efficiencies of ordinary people and military personnel (5). In the early stages, there are no symptoms in the eyes of the victims, but the eyes should be washed in the first 10-15 minutes. Because the eyes reacted immediately to sulfur mustard, this reaction is irreversible and washing after this time is not beneficial (10). Specialists recommend washing with a large volume of accessible compounds and solutions such as tap water, normal saline, sodium bicarbonate 1% or 1.5%, saturated boric acid, diluted sodium hypochlorite solution, potassium permanganate, olive oil, as well dichloramine-t 0.5% and liquid albolene. The available studies have not compared and evaluated which solution is more effective than another (5, 10, 44). Another noteworthy point is that; for the eye pads, gauzes and bandages must not be used because it causes a worse condition for the eyes. This action will increase the temperature in the damaged eyes and cause to lesions (13). Moreover, do not use local anesthetic drops in the examination of healthy and damaged corneas (10, 13). People with photophobia is suggested to use sunglasses (13). In general, the use of topical steroids reduces the chemosis and corneal epithelial edema, but if corneal epithelial defect is proven, the use of topical steroids should be avoided (33).

The victims suffer from ocular pain due to spasm of the ciliary muscles and posterior synechiae, the use of mydriatic drops such as cyclopentolate and atropine to reduce spasm and pain prevents posterior synechiae. These victims may have a secondary bacterial infection. Antibiotics, such as sulfacetamide, gentamycin, neomycin. Chloramphenicol, polymyxin-B sulfate may be used. Topical anti-glucoma medications are prescribed to control intraocular pressure (IOP) (22, 45). One of the victim's eye’s problems is the formation neovascularization, corneal erosion, corneal ticking and corneal edema. The use of anti-inflammatory drugs, especially in the first hour for a week, prevents symptoms due to the formation of neovascularization. Dexamycin (dexamethasone + neomycin) as an anti-inflammatory and antibiotic reduces the symptoms in the eyelids, conjunctiva and cornea. The severity of corneal injuries is decreased about 50% with the administration of dexamycin, as well as the thickness of the cornea is reduced, but dexamycin has no effect on corneal erosion. In general, dexamycin and other anti-inflammatory drugs have been emphasized by ophthalmologists due to reduction in corneal thickness, edema and decrease of neovascularization (23). Tetracycline and doxycycline cause to limit neutrophil collagenase and epithelial gelatinase gene expression, as well as prevent the degradation of alpha-antitrypsin and take in reaction oxygen species, in addition to their antimicrobial properties, they prohibit the activity of matrix metalloproteinase (MMP).Their anti-inflammatory properties reduce acute and delayed injuries, moreover, they are useful in treating moderate to severe eye injuries (23, 45).

**2)** **Surgical Interventions**

Victim's problems with eye injuries because of contact with sulfur mustard are divided into two categories; acute, chronic and delayed. Clinical findings in acute conditions include; sore eyes, lacrimation, burning sensation, edematous, obvious blepharospasm of eyelids, severe conjunctivitis, erosion and opacity of the cornea. Although all of symptoms can be returned to a completely normal condition after a few weeks, it is important to notice that in chronic and delayed lesions, eye problems progress like reduced visual acuity and even blindness. In severe cases, victims suffer from mustard gas keratopathy (MGK), which is one of the most significant chronic and delay complications, and is seen in 0.5 to 1 percent of victims.It is often expanded, and complicated treatment (27, 46, 47) . Lack or disability in the corneal sensation, corneal epithelium erosion that is recurrent/persistent, damaging to the limbal vessels, especially in the nasal or temporal region, as well as irregular and thinning of the cornea and the formation of neovascularization are eye damages by sulfur mustard poisoning. This damage has reached the depth of corneal layers, which its severity is in the anterior and middle part than in the posterior part. Researchers reported the development of descemetoceles and sometimes corneal perforation (25, 28, 46).

● **2-1) Tarsorrhaphy**

Tarsorrhaphy is the joining part or all of the upper and lower eyelids, for the eyes to partially or completely close. In general, tarsorrhaphy will be used to facilitate the recovery of corneal epithelial disorders, or prevent the cornea from being exposed to harmful factors, as well as inherent complications. Temporary tarsorrhaphy helps to the recovery of the cornea, or protects the cornea in short- term, and permanent tarsorrhaphy protects the cornea and prevents potential risks and damages in long-term (48, 49).

To prevent the progress of corneal thinning in nasal or temporal region with or without persistent epithelial defects (PEDs), medial or lateral tarsorrhaphy can be used (22, 27, 50). This method reduces the symptoms of chronic eye irritation and dry eye and develops the healing process. It is usually done after stem cell transplantation or corneal transplantation (22).

● **2-2) Human Amniotic Membrane Transplantation**

The use of an amniotic membrane was first proposed and used by De Rotth in conjunctival surgery in the 1940.It is also reintroduced in 1995 by Vin and Tseng in eye surgery (51). Histologically, the amniotic membrane has a single epithelial layer, its basal membrane is thick, and it is an avascular stroma (52). The usefulness of the amniotic membrane is thinner, and is tolerable for patients. It is the deeper layer of embryo membrane. Its anti-angiogenic, anti-inflammatory, anti-scarring, anti-microbial and anti-fibrotic properties are prominent. As mentioned, it lacks blood vessels and has multi layers. Due to lack of antigenicity, the amniotic membrane is never rejected after transplantation. If the cryopreservation is done well, the inflammation and the formation of neovascularization are postponed for a long time (53, 54).

Amniotic membrane (AM) has abundant benefits. The existing amniotic membrane capability provides a suitable substrate for growth, migration and adhesion of epithelial corneal and conjunctional cells (53, 54).It can be replaced as a graft in damaging ocular surface stromal matrix or in preventing unwanted inflammation in damaging the eye surface as a patch (dressing) is used. It should be noted that it can be used in combination (55). Whenever limbal epithelial stem cells are degraded and/or limbal stroma (niche) is ineffective, limbal stem cell deficiency can occur(50). If there is a persistent epithelial defects (PEDs) with a partial limbal stem cell deficiency (LSCD), transplantation of the human amniotic membrane will be one of the applications. Studies have shown that limbus involvement in victims’ ranges from 120 degrees to approximately 360 degrees. Eventually, with these measures in the eye, there will be a smooth and stable corneal epithelial surface, lack of erosion or persistent epithelial defect, as well as a loss of stromal opacity and the neovascularization. It is important to note that, in severe or total LSCD, amniotic membrane transplantation will not be beneficial (22, 56). Importantly, ophthalmologists do not use sutures in defects in the corneal epithelial, but use fibrin glue instead of sutures. Because the use of sutures cause the growth of the epithelium under the amniotic membrane (56, 57).One of the problems that occurs in victims is photophobia, when the victims have severe eye irritation, also in the examination of the cornea, lipid deposits are seen. Subsequently, superficial keratectomy with amniotic membrane transplantation is recommended by specialists. This action is very useful (23). According to a doctor’s diagnosis, in the treatment of deep corneal and scleral ulceration, frequent amniotic membrane transplants are performed (51).

**● 2-3) Stem Cell Transplantation**

The cornea has stem cells that preserve the visual transparency by maintaining the integrity of the cornea epithelium. These cells, called limbal stem cells (LSCs), are mainly located in the Limbus, a narrow area in the cornea, directly nearby to the conjunctiva. Corneal integrity depends on the proper function of these cells. The normal limbus and action of the limbal stem cells will be a barrier to the invasion of the conjunctival epithelial cells against the cornea (58, 59).

Victims of chemical weapons carrying mustard gas have eye problems such as irritation, redness, dry eyes, tearing of the eyes with persistent epithelial defects (PEDs), focal thinning and corneal ulcers. Histological studies also suggest the formation of stromal neovasculation, loss of keratocytes, endothelial cells, and the deposition of lipid and amyloid. This is due to keratitis caused by mustard gas, which requires stem cell transplantation (22, 39, 47, 50). Surgical procedures for total limbal stem cell deficiency (LSCD) in one eye and in both eyes are different; whenever this defect is in one eye, it is necessary to perform limbal conjunctival autograft transplantation. But if this defect is in both eyes, limbal epithelial stem cells are transplanted with allogeneic origin (60). Limbal stem cell harvesting is from two sources; 1- Family members include parents, brother and sisters, or children. 2- Cadaveric eye. The first is known as living-related conjunctival -limbal allograft (lr-CLAL) and the second is known as keratolimbal allograft (KLAL) (61).

These two methods are comparable in term of genetics, availability, and the presence of stem cells; tissue harvested from family members is fresher and genetically closer than from cadaver. But cadavor grafts (KLAL grafts) are more available and they have more stem cells than family member’s grafts (lr-CLAL grafts). Further, they have weaker stem cells and in chronic situation, transplant rejection is often more likely (22). In surgical technique, it is important to choose the surgical site, the thinnest part of the peripheral cornea with epithelial defect, adjacent to the limbal area is the best location (22, 50). In LSCD, if the severity of the quadrants involvement is determined, and damage to the cornea is small, bilateral and asymmetric, in surgical technique will not require a 360- degree coverage of the limbal area by graft (50). Various immunosuppressive agents, topical and systemic steroids are used to prevent corneal transplant rejection. Prominent and well-known agents include; Prednisone 0.5 mg/kg/day P.O, Cyclosporin A 2.5 mg/kg/day P.O, Tacrolimus (FK506) 0.2 mg/kg/day P.O, Mycophenolate mofetil 2gr/day P.O (62).

● **2-4) Corneal Transplantation**

Eduard Zirm fulfilled the first successful human corneal allograft in 1905.At the present time, more than 100000 corneal transplantations are carried out due to different eye disorders each year in the world (63, 64). After the ophthalmologists evaluate the LSCD, if the deficiency is not severe and central corneal is opacity, which reduces visual acuity, keratoplasty is required. It is performed in two methods: penetrating keratoplasty (PKP) or lamellar keratoplasty (LKP). However, these specialists now tend to perform laminar keratoplasty for selective removal and replacement of the cornea layers of these victims (27, 29, 65, 66). Studies of different layers of the cornea in chronic and delayed keratitis and mustard gas have been observed; the abnormality is seen in all layers, but the severity of the abnormality in the anterior to middle part is more than in the posterior part (47). When a full- thickness graft is needed, the lesions are deep (22). It is possible that the cornea may be perforated due to contact with mustard gas. If the cornea becomes too thin and a large descemetoceles occurs, the patients will undergo tectonic PKP, but if the descemetoceles is small, tectonic LKP will be performed (22, 28).

New methods that can be used in patients with normal endothelium and deep posterior stromal ulcers that have not penetrated the stromal membrane, deep anterior lamellar keratoplasty (DALK) or deep lamellar keratoplasty (DLK) are recommended. These procedures are performed at the deepest level. The reduction of the wound, its thickness in the posterior part is the same, appropriate thickness for tissue donation, the edge of the graft are regular, and the same stretch of the suture are the advantages of these methods (67).

The aims of DALK method are to maintain the entire host's endothelium, remove and return the complete or almost complete corneal stroma (68).Less rejection of endothelial graft (69), minimal surgical trauma to the host endothelium and cell count(68, 70), greater ease and convenience of vision rehabilitation (71),in addition to less complication during surgery and after surgery are some of the benefits of DALK to PKP (68). To protect the density of endothelial cell, DALK is better than PKP. The existing document suggests that the survival time of graft DALK is longer and softer than PKP, because it is an extraocular surgical technique (67). There are different beliefs about surgical technique in simultaneous corneal transplantation and limbal stem cell transplantation, but most ophthalmologists prefer corneal transplantation 3 months after limbal stem cell transplantation. There are many reported and several clinical findings that exposure to mustard gas causes damage to the surface of the eye and keratopathy, so corneal transplantation is associated with high risk. Therefore, the experience of ophthalmologists and reputable published sources states; delay in corneal transplantation will reduce the chance of rejection (22, 50)

## Conclusion

Synthetic compounds of mustard, including sulfur mustard, have been used frequently in the nineteenth and twentieth centuries and the present century and have destructive effects on the body systems of ordinary people and military forces. The mechanisms mentioned by specialists related to this poisoning are as follows: damage to the DNA, an increase in the ROS level, on the other hand, the inactivation of factors that play a significant role against the ROS, such as glutathione (when exposed to sulfur mustard, glutathione depletion occurs). These processes cause damage to various organs in the body. These effects are on various systems, but their vulnerability to the skin, the respiratory system, especially the eye, require careful and accurate managements. 

As noted, in the case of eye problems, victims suffer from acute, chronic and delayed symptoms. These victims need immediate and medical care. If these measures are not successful, surgery will be done. Victims and their countries will be involved in a number of problems in the long time. Therefore, authors and lovers of humanity and nature wish to stop responsible systems for the production and use of chemical weapons, also mass massacre. This behavior is in the hands of all countries in the world, especially the individuals. We hope so to see peaceful coexistence of all nations in the world. 
